# The Role of the BMP Signaling Antagonist Noggin in the Development of Prostate Cancer Osteolytic Bone Metastasis

**DOI:** 10.1371/journal.pone.0016078

**Published:** 2011-01-13

**Authors:** Chiara Secondini, Antoinette Wetterwald, Ruth Schwaninger, George N. Thalmann, Marco G. Cecchini

**Affiliations:** Urology Research Laboratory, Department of Urology and Department of Clinical Research, University of Bern, Bern, Switzerland; Baylor College of Medicine, United States of America

## Abstract

Members of the BMP and Wnt protein families play a relevant role in physiologic and pathologic bone turnover. Extracellular antagonists are crucial for the modulation of their activity. Lack of expression of the BMP antagonist noggin by osteoinductive, carcinoma-derived cell lines is a determinant of the osteoblast response induced by their bone metastases. In contrast, osteolytic, carcinoma-derived cell lines express noggin constitutively. We hypothesized that cancer cell-derived noggin may contribute to the pathogenesis of osteolytic bone metastasis of solid cancers by repressing bone formation. Intra-osseous xenografts of PC-3 prostate cancer cells induced osteolytic lesions characterized not only by enhanced osteoclast-mediated bone resorption, but also by decreased osteoblast-mediated bone formation. Therefore, in this model, uncoupling of the bone remodeling process contributes to osteolysis. Bone formation was preserved in the osteolytic lesions induced by noggin-silenced PC-3 cells, suggesting that cancer cell-derived noggin interferes with physiologic bone coupling. Furthermore, intra-osseous tumor growth of noggin-silenced PC-3 cells was limited, most probably as a result of the persisting osteoblast activity. This investigation provides new evidence for a model of osteolytic bone metastasis where constitutive secretion of noggin by cancer cells mediates inhibition of bone formation, thereby preventing repair of osteolytic lesions generated by an excess of osteoclast-mediated bone resorption. Therefore, noggin suppression may be a novel strategy for the treatment of osteolytic bone metastases.

## Introduction

Skeletal metastasis is a common clinical manifestation in advanced-stage patients suffering from prostate cancer (CaP) [1], [2] and mammary cancer (CaM) [3]. Bone metastases are the most important cause of morbidity in these patients, with pain and complications, including pathological fractures, spinal cord and nerve compression requiring analgesia, irradiation and orthopedic surgery, all associated with substantial costs [4].

At the metastatic site, tumor cells perturb the physiological bone homeostasis controlled by osteoblasts and osteoclasts. CaM bone metastases tend to elicit an osteolytic response, whereas CaP metastases are prevalently associated with an osteosclerotic reaction [5], [6]. Both types of lesions compromise the skeletal integrity and eventually lead to pathological fractures.

The exact mechanisms determining the osteolytic and osteosclerotic lesions in bone metastases are not clearly defined yet. The prevailing concept indicates that cancer cells secrete an excess of paracrine factors stimulating directly or indirectly osteoclast or osteoblast recruitment, thereby leading to unbalanced excess of bone resorption or formation, respectively [7], [8].

It is widely accepted that the osteolytic reaction in bone metastasis results from an excess of osteoclast-mediated bone resorption. Cancer cells release paradigmatic “osteolytic” cytokines, such as parathyroid hormone-related protein (PTHrP), receptor activator of NF-B ligand (RANKL), interleukin-8 (IL-8) and colony stimulating factor-1 (CSF-1), directly or indirectly responsible for the increase in osteoclast recruitment, activity and survival. Subsequent release of growth factors from the bone matrix fuels cancer cell growth, which in turn further stimulates bone resorption, thus perpetuating the process and establishing a “vicious cycle” [5], [9]. This hypothesis provides the rationale for inhibition of bone resorption as therapeutic interference with growth progression in osteolytic bone metastasis. However, pharmacologic inhibition of bone resorption has only a minimal or no positive impact on the healing of osteolytic lesions [10]. This strongly suggests that, besides an increase in osteoclast-mediated bone resorption, other mechanism(s) contribute to osteolysis.

The osteolytic lesion in multiple myeloma (MM) is not only the result of an osteoclast-mediated increase in bone resorption [11], but also of an uncoupling of the bone remodeling process determined by a decrease in osteoblast-mediated bone formation [12], [13]. Several antagonists of the Wingless (Wnt) signaling pathway, such as Dickkopf-1 (Dkk-1), secreted Frizzled-related protein (sFRP) -1 and -2, are over-expressed by MM cells and may contribute to the inhibition of Wnt-mediated osteoblast recruitment and, therefore, to repression of bone formation [11], [14], [15]. This view is further corroborated by experimental evidence showing that blocking Dkk-1 activity rescues bone formation in animal models of MM [16].

Previously, we have reported that the osteoinductive and osteolytic potential *in vivo* of CaP and CaM cell lines can be defined *in vitro* by their differential expression not only of osteolytic cytokines, but also of the BMP antagonist noggin. Osteoinductive cancer cell lines lack noggin expression, and the functional relevance of this finding was emphasized by showing that noggin forced expression in an osteoinductive CaP cell line abolishes the osteoblast response induced by its bone metastases *in vivo*. Conversely, constitutive noggin expression *in vitro* seems to be the hallmark of osteolytic CaP and CaM cell lines [17]. We then argued that, in analogy to what has been found for Dkk-1 in MM, constitutive noggin release by osteolytic cancer cells might contribute, through inhibition of bone formation, to the osteolytic lesion in bone metastases of solid cancers.

To test this hypothesis, we first explored whether inhibition of bone formation is a constituent of osteolytic lesions induced in bones xenografted with the human CaP cell line PC-3. We then investigated whether short hairpin RNA (shRNA)-mediated noggin suppression in PC-3 cells, constitutively secreting this protein [17], could preserve bone formation in the osteolytic lesions.

The osteolytic lesions in bones xenografted with PC-3 cells showed morphological and histomorphometric parameters of enhanced osteoclast-mediated bone resorption and decreased osteoblast-mediated bone formation. In contrast, bones xenografted with noggin-silenced PC-3 cells were characterized by structural and histological modifications indicative of bone formation/repair activity. Therefore, noggin suppression in the osteolytic cell line PC-3 seems to preserve the bone formation that normally follows bone resorption, as an effect of the “coupling phenomenon”. Conversely, cancer cell-secreted noggin prevents the repair of osteolytic lesions by uncoupling bone formation from the osteoclast-mediated bone resorption, which is stimulated by cancer cell-derived osteolytic cytokines.

## Results

### Silencing of Noggin mRNA and protein expression in the PC-3/F*luc* Cell clone by shRNA

The human osteolytic CaP cell line PC-3, constitutively expressing the BMP antagonist noggin [17], was first transfected with a luciferase-encoding vector to generate *luc*-positive clones. A cell clone, PC-3/F*luc*, was selected based on stable *luc* expression and on gene expression profile *in vitro*, tumor take and bone reaction after intra-osseous inoculation *in vivo* equivalent to those of the parental PC-3 cells (Figure S1).

**Figure 1:**
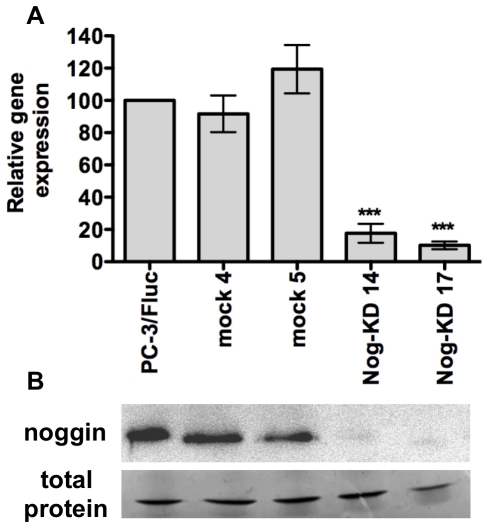
Noggin silencing mediated by shRNA inhibits noggin mRNA and protein expression. **A**. Noggin mRNA expression in PC-3/F*luc* cells, non targeting-vector-transfected clones (mock 4 and 5) and Noggin-KD clones (Nog-KD 14 and 17). The mRNA expression levels (+/− SD) are quantified by real-time RT-PCR and normalized to β-actin as endogenous control; mRNA level in PC-3/F*luc* cells is set as 100%; the mean of 2 to 3 independent experiments is shown. ***P<0.001, Nog-KD clones *versus* PC-3/F*luc* and mock clones. **B**. Noggin protein secreted in the conditioned medium (CM) from PC-3/F*luc*, mock and Nog-KD clones. Equivalent amounts of proteins from concentrated CM were immunoblotted with anti-noggin antibody. Equivalent protein loading was verified by staining with Coomassie blue the total protein content in the CM.

The PC-3/F*luc* clone was subsequently transfected with a combination of three different noggin-specific shRNA or with a non-targeting shRNA to derive noggin knock-down (Nog-KD) and negative control (mock) clones, respectively. Two Nog KD clones (Nog-KD 14 and Nog-KD 17) were selected based on the substantial reduction in noggin mRNA expression, as compared to PC-3/F*luc* cells (Figure 1A). Immunoblot analysis of noggin protein secretion in the culture supernatant from the Nog-KD clones 14 and 17 showed a reduction of 93% and 98%, respectively (Figure 1B). Mock 5 clone showed an increase in noggin mRNA expression. However, noggin protein expression in this clone and in mock 4 was not affected.

### Cell proliferation and expression of osteotropic factors of the Noggin-silenced PC-3/F*luc* cells *in vitro*

**Figure 2:**
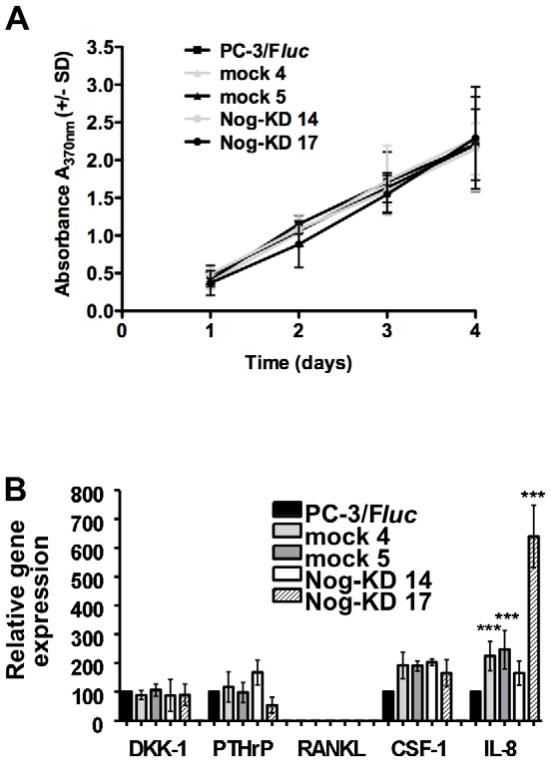
Noggin silencing has no effect on cell proliferation and affects only minimally the expression of osteotropic factors, *in vitro*. **A**. *In vitro* proliferation of mock and Nog-KD clones was measured by BrdU incorporation for 4 days and compared to PC-3/F*luc* cells. Average values of 3 independent experiments performed in quadruplicate wells (+/− SD). **B**. Expression of Dkk-1, PTHrP, RANKL, CSF-1 and IL-8 mRNA. mRNA expression levels (+/− SD) are quantified by real-time RT-PCR and normalized to β-actin as endogenous control. The mRNA expression level in PC-3/F*luc* cells is set as 100%; the mean of 2 to 3 independent experiments is shown. ***P<0.001, mock and Nog-KD 17 clones *versus* PC-3/F*luc* and Nog-KD 14 clones.

Cell proliferation *in vitro* of the mock and Nog-KD clones was not affected, as compared to parental PC-3/F*luc* (Figure 2A).

In order to verify whether noggin silencing would affect mRNA expression of relevant osteotropic factors, the expression of Dkk-1, PTHrP, RANKL, CSF-1 and IL-8 was also investigated (Figure 2B). The expression of Dkk-1 and PTHrP was not modified in all transfected clones, as compared to the PC-3/F*luc*. Consistently with previous investigations in parental PC-3 cells [17], RANKL expression was undetectable in PC-3/F*luc*, mock and Nog-KD clones. CSF-1 was moderately up-regulated in all mock and Nog-KD clones, although the elevation did not reach significance. IL-8 expression was significantly higher in the mock clones and, especially, in Nog-KD 17.

It has been shown in osteoblasts *in vitro* that expression of noggin is BMP-dependent, indicating that a probable feedback mechanism is necessary to maintain a BMP/noggin balance, thereby limiting the BMP action on these cells [23]. A similar feedback has also been reported in some prostate cancer-derived cell lines [17], [24]. The mRNA expression of BMP-2,-3,-6 and -7 was not modified in both Nog-KD clones as compared to parental PC-3, while BMP-4 mRNA was undetectable in both parental and Nog-KD cells (not shown). Therefore, noggin silencing in PC-3 cells does not alter either the level or the spectrum of their BMP expression.

### Effect on bone *in vivo* of Noggin-silenced PC-3/F*luc* cells

**Figure 3:**
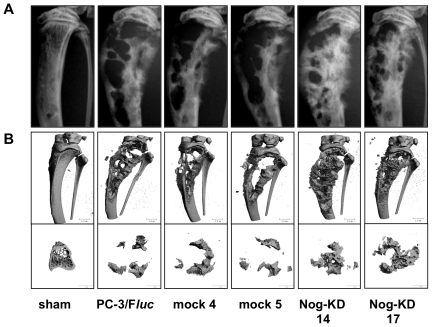
Noggin silencing promotes increase in radiodensity and partial bone repair in advanced osteolytic lesions. **A**. Representative images of radiography and **B**. 3-D reconstruction (µ-CT) of tibiae (top) and of 1 mm thick cross sections (bottom) of sham-operated and cancer cell-xenografted tibiae at day 25 after intra-osseous inoculation.

A first experiment was terminated at day 25 after injection for all groups of animals, while in a following experiment the observation period for the Nog-KD clones was prolonged to 33 days. In both experiments the radiographic analysis detected the onset of an equivalent osteolytic reaction in all groups of cancer cell-bearing bones as early as day 14 after tumor cell xenografting (not shown). At day 21 all xenografted tibiae still showed a similar number and extension of osteolytic lesions, and an enlargement of the bone shaft, as compared to sham-operated tibiae. However, in tibiae xenografted with Nog-KD clones the residual bone interposed between areas of osteolysis showed some evidence of a more radio-dense aspect (Figure S2). In the first experiment, both the radiography and the µ-CT reconstruction performed at the conclusion of the experiment (day 25) indicated further development of predominantly osteolytic lesions in PC-3/F*luc-* and mock clone-bearing tibiae (Figure 3A and B). In contrast, in the tibiae xenografted with the Nog-KD clones an evident increase in radiodensity of the residual bone and, in addition, development of bone spiculae projecting outside the cortex was invariably observed (Figure 3A and B). In the second experiment the observation period was prolonged in order to allow progression of the bone response. The animals inoculated with the PC-3/F*luc* clone needed to be sacrificed at day 23 due to severe osteolysis of the xenografted bones and swelling of the limb, and to signs of pain and distress. In contrast, bones xenografted with the Nog-KD clones showed a more preserved structural integrity and a less pronounced swelling of the limb, with no signs of pain and distress. Accordingly, the animals xenografted with Nog-KD 17 and Nog-KD 14 clones were kept for a period of 30 and 33 days, respectively. Consistently with the evolution of the bone response observed in the first experiment, tibiae xenografted with Nog-KD clones showed enhanced radiodensity of the residual bone and pronounced bone spiculae projecting outside the cortex (Figure S3A and B).

### Modification of bone structural parameters induced *in vivo* by Noggin-silenced PC-3/F*luc* cells

**Figure 4:**
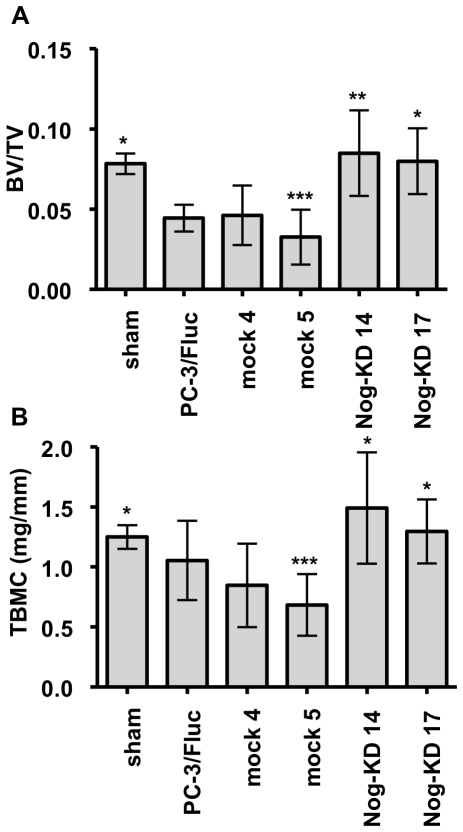
Noggin silencing corrects the alterations of bone structural parameters in osteolytic lesions. **A**. The ratio of bone volume over total volume (BV/TV, +/− SD) was determined by µ-CT at day 25 after tumor cells inoculation; *n* = 6–7 animals for each experimental group. ***P<0.001, mock 5 *versus* Nog-KD clones and sham; **P<0.01, Nog-KD 14 *versus* PC-3/F*luc* and mock 4; *P<0.05, sham and Nog-KD 17 *versus* PC-3/F*luc* and mock 4. **B**. Total bone mineral content (TBMC; mg/mm +/− SD) was measured by pQCT at day 25 after tumor cells inoculation; *n* = 6–7 animals for each experimental group. ***P<0.001, mock 5 *versus* Nog-KD 14; *P<0.05, sham and Nog-KD 17 *versus* mock 5, and Nog-KD 14 *versus* mock 4.

Quantitative µ-CT analysis confirmed that the tibiae xenografted with the PC-3/F*luc* and mock clones had significantly lower BV/TV ratio than the sham-operated ones. On the contrary, the tibiae inoculated with the Nog-KD clones had higher BV/TV ratio than with PC-3/F*luc* and mock clones and were not different from the sham-operated ones (Figure 4A).

Total bone mineral content measured by pQCT was lower in the tibiae xenografted with the PC-3/F*luc* and mock clones, as compared with the sham-operated ones, but significance was reached only for mock 5-xenografted bones. In contrast total bone mineral content in the Nog-KD clones-xenografted tibiae was higher than in PC-3/F*luc* and mock clones-xenografted ones, but reached significance only when compared to the mock clones (Figure 4B).

### Histomorphometry

**Figure 5:**
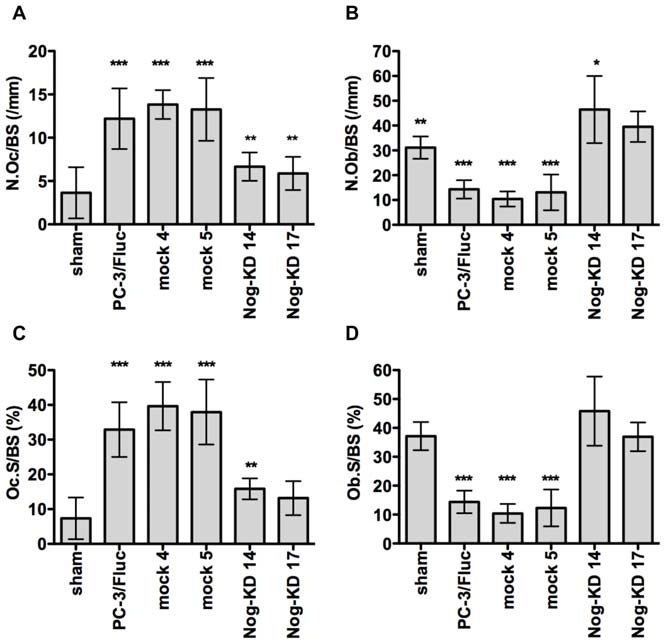
Noggin silencing normalizes the bone histomorphometric indexes of bone formation and resorption in osteolytic lesions. **A**. Number of osteoclasts (N.Oc/BS; /mm, +/− SD) on the endosteal surface in trabecular and cortical bone of sham-operated tibiae or on residual bone adjacent to cancer cells of tibiae xenografted with PC-3/F*luc*, mock and Nog-KD clones. *n* = 6–7 animals for each experimental group. ***P<0.001, PC-3/F*luc* and mock clones *versus* sham, and mock clones *versus* Nog-KD 17; **P<0.01, Nog-KD 17 *versus* PC-3/F*luc,* and Nog-KD 14 *versus* PC-3/F*luc* and mock clones. **B**. Number of osteoblasts (N.Ob/BS; /mm, +/− SD) on the endosteal surface in trabecular and cortical bone of sham-operated tibiae or on residual bone adjacent to cancer cells of tibiae xenografted with PC-3/F*luc*, mock and Nog-KD clones. *n* = 6–7 animals for each experimental group. ***P<0.001, PC-3/F*luc* and mock clones *versus* Nog-KD clones, and mock 4 *versus* sham; **P<0.01, sham *versus* PC-3/F*luc* and mock 5; *P<0.05, Nog-KD 14 *versus* sham. **C**. Percentage of endosteal surface in cortical and trabecular bone occupied by osteoclasts (Oc.S/BS; %, +/− SD) in sham-operated and cancer cell-xenografted tibiae. *n* = 6–7 animals for each experimental group. ***P<0.001, mock clones *versus* sham and Nog-KD clones, and PC-3/F*luc versus* sham and Nog-KD 17; **P<0.01, Nog-KD 14 *versus* PC-3/F*luc*. **D**. Percentage of endosteal surface in cortical and trabecular bone occupied by osteoblasts (Ob.S/BS; %, +/− SD) in sham-operated and cancer cell-xenografted tibiae. *n* = 6–7 animals for each experimental group. ***P<0.001, PC-3/F*luc* and mock clones *versus* sham and Nog-KD clones.

In the tibiae xenografted with PC-3/F*luc* and mock clones there was a significant increase in osteoclast number and percentage of surface covered by osteoclasts, as compared to the sham-operated tibiae (Figure 5A and C). This was accompanied by a significant decrease in osteoblast number and percentage of surface covered by active osteoblasts (Figure 5B and D).

Tibiae xenografted with the Nog-KD clones showed significantly lower number of osteoclasts and percentage of surface covered by osteoclasts than those xenografted with PC-3/F*luc* and mock clones (Figure 5A and C). Conversely, they showed a significantly higher number of active osteoblasts and percentage of surface covered by osteoblasts than those inoculated with PC-3/F*luc* and mock clones. In Nog-KD clones-xenografted tibiae the number of osteoblasts was higher than in the sham-operated tibiae, but this increase was significant only for Nog-KD 14 (Figure 5B and D). Despite the fact that the mRNA expression of IL-8, known to stimulate osteoclast recruitment, was significantly up-regulated *in vitro* in the Nog-KD 17 clone, as compared to the Nog-KD 14 clone, there was no obvious difference in the osteoclast number between the bones xenografted with the two Nog-KD clones. This suggests that the difference in IL-8 mRNA expression was functionally irrelevant.

### Growth characteristics *in vivo* of Noggin-silenced PC-3/F*luc* cells

**Figure 6:**
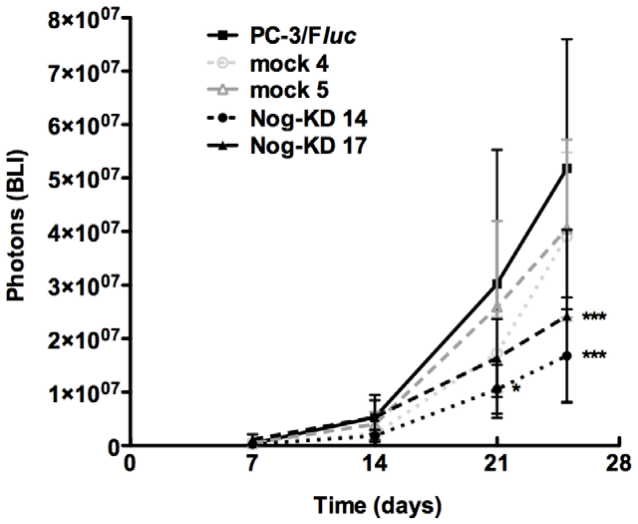
Noggin silencing limits growth of PC-3/F*luc* cells in bone xenografts. Bioluminescent signal (photons +/− SD) emitted from the cancer cell-xenografted tibiae was quantified at day 7, 14, 21 and 25 after intra-osseous inoculation of tumor cells; *n* = 6–7 animals for each experimental group. ***P<0.001, Nog-KD clones *versus* PC-3/F*luc* at day 25; *P<0.05, Nog-KD 14 *versus* PC-3/F*luc* at day 21.

Tumor take was 100% for all xenografted clones. Weekly monitoring and quantification of intra-osseous tumor growth by BLI revealed that, in both experiments, noggin suppression in cancer cells had a moderate impact on their proliferation *in vivo*. Initially, the Nog-KD clones grew similarly to PC-3/F*luc* and mock clones. However, their growth progressively slowed down and they could not reach the same tumor burden as for PC-3/F*luc* and mock clones (Figure 6). In the second experiment, where tumor growth was monitored for longer periods, a growth arrest was even observed (Figure S3C).

### Metastatic potential of Noggin-silenced PC-3/F*luc* cells

In order to investigate whether noggin silencing influences the metastatic ability of PC-3 cells, systemic metastases were induced by injection into the left cardiac ventricle.

The kinetics of development and the number of bone metastases per mouse 28 days after intra-cardiac injection of Nog-KD 17 cells did not differ from that induced by PC-3/F*luc* and mock clones (not shown). After intra-cardiac injection of PC-3/F*luc* cells systemic bone metastases develop asynchronously at variable bone sites, making difficult a direct comparison of the radiographic aspect and growth progression of metastatic lesions at the same bone site among different animals. Furthermore, PC-3/F*luc* cells, like the parental PC-3, almost invariably metastasize to the jaw, thereby impairing the nutritional status of the animals and, consequently, limiting the length of the experimental observation. These drawbacks prevented us to verify whether in this model the bone response and the tumor growth induced by the Nog-KD clone progress equally to those observed in the intra-osseous model.

## Discussion

Here we show for the first time that in the PC-3 xenograft model of osteolytic bone metastasis the increase in osteoclast number is associated with impairment in osteoblast number and activity. This indicates that the osteolytic lesion is not only the result of increased bone resorption, but also of an additional inhibition of bone formation. We also demonstrate that shRNA-mediated suppression of the constitutive expression of the BMP antagonist noggin in PC-3 cells, without interfering with the host microenvironment–derived noggin, restores the osteoblast number and bone formation in bone lesions induced by these cells. Accordingly, the bone response is converted from a purely osteolytic to a mixed osteoblastic/osteolytic one. Collectively, these results provide novel evidence strongly suggesting that noggin secretion by CaP cells mediates the inhibition of the osteoblast recruitment/activity. The resulting inhibition of bone formation prevents the repair of the osteolytic lesion generated by cytokine-stimulated, osteoclast-mediated bone resorption. Consistently with this notion, noggin may represent a potential therapeutic target in osteolytic CaP bone metastasis.

The molecular mechanisms governing the osteoblastic and the osteolytic response in bone metastases by solid cancers are subject of intensive investigation. In the osteolytic response, the attention has been predominantly focused on extracellular factors and signaling pathways that mediate the crosstalk between tumor cells and the bone microenvironment leading to the vicious cycle of tumor proliferation and bone resorption [25]. This hypothesis postulates that factors such as PTHrP [26], RANKL [27] and IL-8 [28] are secreted by cancer cells and stimulate osteoclast recruitment and activity. The consequent increase in bone resorption releases matrix-embedded growth factors, such as insulin-like growth factor (IGF) and transforming growth factor beta (TGF-β), which, in turn, promote further cancer cell growth.

The influence of cancer cells on osteoblast recruitment and activity has been studied almost exclusively in MM. Paradigmatic molecules inducing directly osteoblast recruitment and, consequently, bone formation, are members of the BMP and Wnt protein families. Extracellular antagonists are crucial for the modulation of their activities [29]. In a seminal study it has been demonstrated that in MM secretion of the Wnt antagonist Dkk-1 by the neoplastic cells inhibits osteoblast recruitment and activity. Accordingly, the osteolytic lesion in MM is not only the result of increased bone resorption, but also of repressed bone formation [30]. Furthermore, down-regulation of Dkk-1 expression seems to mediate the osteoinductive activity of endothelin-1 (ET-1) [31]. Another extracellular antagonist of the Wnt pathway, sFRP-2, may also contribute to this mechanism of inhibition of bone formation [32].

The modulation of the osteoblast recruitment/activity and the possible contribution of inhibition of bone formation in osteolytic bone metastasis by solid cancers have received little attention. A limited number of quantitative histomorphometric studies in osteolytic metastases by a variety of epithelial cancers have shown that, besides the increase in bone resorption, there is also impairment in bone formation, especially in advanced lesions [33], [34], [35]. It has been suggested that in these lesions bone resorption is uncoupled from the subsequent bone formation phase, which usually follows in normal bone remodeling [36]. This phenomenon may be mediated by a direct, negative influence of cancer cells on osteoblast recruitment, survival and activity, as shown *in vitro* for osteolytic CaM and CaP cell lines [37], [38], [39]. The present investigation, showing that indexes of bone formation are impaired in tibiae xenografted with PC-3 cells, further supports the clinical findings above and strongly suggests that a mechanism of uncoupling bone formation from resorption is also operating in this model. However, the identity of the molecules mediating this inhibitory effect on osteoblasts is yet unknown.

Antagonism of BMP activity by noggin is critical for embryonic chondro-osteogenesis and joint formation [40]. Osteoblast-targeted over-expression of noggin results in osteopenia as the result of impaired osteoblast recruitment [41], [42], indicating that the extracellular modulation of the BMP concentration is also essential in adult life for the control of bone formation during bone remodeling. BMPs and noggin reciprocally induce their expression in osteoblasts [23], indicating that a positive feedback is necessary for maintaining an optimal balance between BMP and noggin concentrations in the bone microenvironment. Bone metastatic cancer cells may interfere with this balance by secreting an excess of either BMPs or noggin. It has been reported that BMP-6 expression positively correlates with CaP progression [43], [44] and that BMP-6 is the BMP primarily responsible for inducing an osteoblast response in mouse models of CaP bone metastasis [17], [45]. However, in one of these reports we demonstrated that, in addition, osteoinductive cancer cells lack secretion of the BMP inhibitor noggin and that noggin forced expression in these cells abolishes their osteoinductive activity *in vivo*. Therefore, an unopposed effect of an excess of BMP-6 locally released by cancer cells is also a determinant of the osteoblast response both in CaP and CaM bone metastasis [17]. Likewise, low expression of the Wnt antagonist Dkk-1 seems to favor the Wnt-induced osteoblast response in CaP bone metastasis [46]. These two studies proved for the first time that in osteoblastic bone metastases the physiological, tight balance between osteoinductive BMP-6 and/or Wnt proteins and their antagonists is tilted toward the first and, thus, favors an abnormal osteoblast response. In contrast, osteolytic CaM cell lines express BMP-2 and -4, while the osteolytic CaP cell line PC-3 expresses BMP-3 [17], [47]. However, both CaP and CaM osteolytic cell lines express low or no BMP-6 [17], [45] and BMP-7 [48], [49] and, most importantly, secrete constitutively relative high amounts of noggin [17]. Thus, in osteolytic bone metastases the balance between BMPs and noggin seems to be altered in a direction opposite to that of osteoblastic bone metastases. The present study proves that this noggin-tilted balance is responsible for the suppression of bone formation and, thus, prevents repair of the osteolytic lesion. Furthermore, the demonstration that specific targeting of the cancer cell-derived noggin preserves bone formation/repair strongly suggests noggin as a major “un-coupling factor” in osteolytic bone metastasis and further emphasizes the relevance of BMP antagonism in pathological bone remodeling.

Here we clearly show that noggin silencing in osteolytic PC-3 cells, although not correcting the bone architecture, restores the bone mass of the xenografted tibiae, as assessed by radiography, µ-CT and pQCT. This effect on the bone mass is the result of normalization or even enhancement of bone formation, as indicated by histomorphometric parameters of osteoblast number and activity. Noggin silencing in PC-3 cells does not alter either the level or the spectrum of their BMP expression. As a result, the physiological BMP/noggin balance in bone could be tilted in favor of first by an excess of cancer cell-derived BMPs, which may explain the trend toward increase in bone formation observed in bone xenografted with Nog-KD PC-3 cells.

The bone repair is evident in advanced bone lesions, suggesting that a time interval is necessary to restore bone formation, consistently with the temporal organization of the physiological bone remodeling (bone coupling). A similar mechanism of restoration of bone coupling has been proposed to explain the reparative bone formation in bone metastasis by solid cancers as an effect of anti-cancer therapy [33], [36], [50].

The positive effect of noggin silencing on bone formation supports our original hypothesis and suggests noggin as one of the essential cancer cell-derived inhibitors of the osteoblast recruitment/activity contributing to the osteolytic lesions in CaP bone metastases. We have chosen a CaP model of osteolytic bone metastasis to be consistent with our previous study demonstrating that, conversely, lack of noggin has a relevant role in the osteoblastic response in CaP bone metastases [17]. However, a predominantly osteolytic phenotype is observed only in a small percentage of CaP bone metastases, while it represents the majority of the CaM metastases [5], [6]. Therefore, in order to extend the relevance of noggin in cancer-mediated osteolysis, it will be important to confirm the present findings in a CaM model of osteolytic bone metastasis. Nevertheless, it has already been shown that inhibition of Dkk-1 by neutralizing antibodies in MM *in vivo* [51], [52], or in CaM cells *in vitro* [53], or by shRNA-mediated interference in CaP cells *in vitro* [54] is also able to restore bone formation suppressed by neoplastic cells. Furthermore, Dkk-1 serum levels are elevated in CaM patients with bone metastases, but not in CaM patients with soft tissue metastases, and it has been suggested that Dkk-1-induced inhibition of bone formation may contribute to the osteolytic lesion in CaM [55]. Collectively, these and the present investigation support the view that cancer cell-derived antagonists of both BMP and Wnt signaling pathways contribute to the severity of the osteolytic process not only in MM, but also in various types of epithelial cancer. The BMP and Wnt signaling pathways clearly cooperate and regulate each other, but the exact hierarchy of these two pathways in stimulating osteoblast recruitment and activity is still debated [56], [57], [58], [59].

In the late phase of the experimental observation, concomitantly to bone repair, noggin silencing in cancer cells also reduces tumor growth. The fact that the growth rate *in vitro* of noggin-silenced clones is not affected suggests that this effect *in vivo* may be exerted through an influence of the bone environment. Restoration of bone formation may physically limit the intramedullary space available for cancer cell expansion [60]. However, in this case cancer cells would most probably invade the surrounding soft tissue through trans-cortical, vascular channels, as it has been observed when bone resorption is inhibited [61], [62]. Most likely, osteoblasts may secrete factors inhibiting tumor growth directly [63]. One of these factors could be BMP-7, which we have previously demonstrated to inhibit tumor growth in animal models of osteolytic bone metastasis [48], [49]. Osteolytic cancer cell-derived noggin may antagonize endogenous, osteoblast-derived BMP-7 and, therefore, allow escape from its inhibitory effect on tumor growth. Alternatively, osteoblasts may affect tumor growth indirectly, acting through an inhibition of osteoclast recruitment [39], [60]. The latter, indirect mechanism is also suggested by the decreased osteoclast number and activity observed in tibiae xenografted with noggin-silenced PC-3 cells. Taken together, these observations indicate that restoration of bone formation may limit growth progression in osteolytic bone metastasis.

The establishment of systemic bone metastases induced by injection into the left cardiac ventricle of noggin-silenced PC-3 cells is not impaired. This suggests that interference with the constitutive secretion of noggin by cancer cells does not affect the early steps of the bone metastatic colonization (adhesion and extra-vasation) and, especially, the growth initiation by the osteolytic process.

It has been previously reported that exogenous noggin inhibits BMP-2- and BMP-4-induced invasion and migration of PC-3 cells, *in vitro*, and that noggin over-expression in the same cells limits their expansion and osteolytic activity, *in vivo* [64]. This finding is apparently in contradiction with our present study, showing that noggin silencing, rather than over-expression, limits tumor growth and osteolysis in the same PC-3 bone xenograft model. Most likely, noggin over-expression in PC-3 cells antagonizes the overall BMPs secreted by both cancer cells and the bone microenvironment and, accordingly, interferes not only with their direct, stimulatory effect on the invasive capacity of PC-3 cells, but also with their normal, stimulatory role in bone formation. In contrast, noggin silencing in PC-3 cells targets exclusively the noggin of cancer cell origin, thereby eliminating its interference with the physiological BMP/noggin balance within the bone microenvironment. Accordingly, noggin silencing seems to be a more targeted mean to understand the role of noggin in osteolytic bone metastasis.

**Figure 7:**
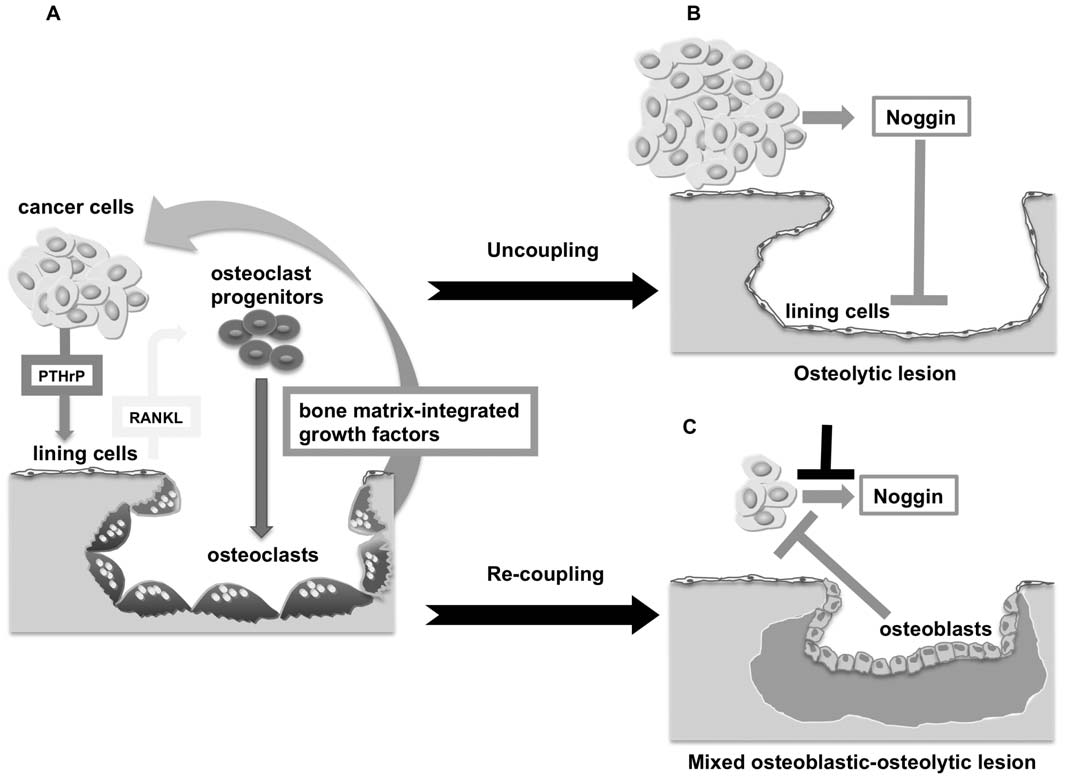
Model of osteolysis integrating a dual mechanism of stimulated bone resorption and “uncoupled” bone formation. The osteolytic lesion is the net result of two different mechanisms leading to uncoupling of normal bone remodeling: **A**. Excess of bone resorption, consequent to enhanced osteoclast recruitment and activity induced by cancer cell-derived osteolytic cytokines. The subsequent release of bone matrix-integrated growth factors further stimulates cancer cell growth. **B**. Suppression of bone formation, consequent to interference with osteoblast recruitment and activity induced by cancer cell-derived noggin. **C**. Noggin silencing in cancer cells re-establishes osteoblast recruitment and activity, and, thus, “bone coupling”, with consequent bone formation/repair. As an effect of the rescued osteoblast activity, tumor progression is contained by yet unidentified osteoblast-derived factors.

Based on the present results we postulate a revised model for the pathogenesis of osteolytic bone metastasis by solid cancers, where the osteolytic lesion is not only the result of excess of bone resorption induced by cancer cell-derived factors stimulating osteoclast recruitment and activity, but also of uncoupling bone formation through noggin constitutively secreted by cancer cells. Conversely, suppression of cancer cell-derived noggin restores the bone formation phase, which is physiologically coupled to the initial phase of bone resorption. As a result of this anabolic effect, tumor progression is contained by yet unidentified, osteoblast-derived factors (Figure 7). Thus therapeutic neutralization of noggin may not only favor bone repair, but also control bone metastatic growth.

Targeting of the RANK/RANKL axis has been proven to successfully inhibit osteolysis in numerous animal models of bone metastasis [65] and this evidence has contributed significantly to the strategy to develop a humanized monoclonal antibody against RANKL for treating osteolytic bone disease [66]. The evidence presented in this investigation, suggesting that a dual mechanism of stimulated bone resorption and inhibited bone formation is responsible also for the osteolytic lesion by solid cancers, provides the rationale for a combinatorial targeting of both, the RANK/RANKL axis and the BMP antagonist noggin for a more effective control of bone metastatic growth.

## Materials and Methods

### Ethics statement

Experimental animal protocols, anesthesia, surgical procedure and post-surgical analgesia were approved by the Committee for Animal Experimentation and the Veterinary Authorities of the Bern State (Permit Number: 15/07). Mice were housed in individual ventilated cages in strict accordance to the Swiss Guidelines for the Care and Use of Laboratory Animals. Autoclaved water and sterile mouse chow were provided *ad libitum*. Animals xenografted with human cancer cells were carefully monitored for signs of pain or distress, loss of body weight and radiological signs of severe osteolysis and imminent risk fracture. When any of these signs appeared or at the end of the experimental period, the mice were sacrificed by CO_2_ euthanasia.

### Animals

BALB/c nude mice were purchased from Charles River France (L'Arbresle, France) and they were 7-8 weeks old when used for the intra-osseous inoculation of tumor cells and 5 weeks old for the intra-cardiac injection. For surgical manipulation, mice were anesthetized as described previously [18].

### Cell lines and cell culture

The osteolytic human CaP cell line PC-3 (ATCC/LGC Promochem, Molsheim, France) was grown in Dulbecco's modified Eagle's medium supplemented with 10% FBS (Biochrom AG, Berlin, Germany) and antibiotics. Cells were shown to be free of mycoplasma by PCR (Venor GeM, Minerva Biolabs GmbH, Berlin, Germany).

### PC-3 cell-derived clones

For *in vivo* tracking, PC-3 cells were permanently transduced with the firefly luciferase (F*luc*) using a self-inactivating lentiviral vector [19] and cloned by limiting dilution. The PC-3/F*lu*c clone was electroporated (Nucleofector, Amaxa, Lonza, Verviers, Belgium) with either the combination of three vectors containing shRNA targeting the noggin transcript or a non-targeting sequence (SureSilencing, SABiosciences, LucernaChem, Lucerne, Switzerland) to generate Noggin knock-down (Nog-KD) and negative control (mock) clones, respectively. The sequences of the shRNA used are listed in Supporting Information (Table S1). Individual stably transfected clones were derived by selection with Puromycin (1 ug/ml) and Neomycin (200 ug/ml) (Sigma-Aldrich, Buchs, Switzerland).

### Generation of conditioned media

Cells were seeded at a density of 2.5×10^4^ cells/cm^2^ in 10 cm culture dishes. After 1 day, the medium was replaced with 10 ml serum-free medium and the cells were cultured for further 48 hours. The cell-conditioned media (CM) were centrifuged and stored at –20°C for later use. The cell number was determined and the volume of CM was adjusted for differences in cell density between samples. CM were dialyzed against serum-free medium and concentrated 10-fold by lyophilisation.

### Cell proliferation assay

Cells were seeded at a density of 10^4^ cells/cm^2^ in microtiter plates and cultured for 4 days. Cell proliferation was determined by measuring bromodeoxyuridine (BrdU) incorporation with a colorimetric ELISA (Cell Proliferation ELISA, BrdU; Roche Diagnostics, Rotkreuz, Switzerland) according to the manufacturer's protocol.

### Real-time polymerase chain reaction (PCR)

Total RNA extraction from subconfluent cultures of PC-3/F*lu*c cells and the various clones was performed with RNeasy (Qiagen, Hombrechtikon, Switzerland). Reverse transcription was performed with M-MLV-RT (Promega, Wallisellen, Switzerland) and random primers (Roche Diagnostics). Human-specific real-time PCR primers and probes (Applied Biosystems, Rotkreuz, Switzerland) are listed in Supporting Information (Table S2).

### Immunoblotting

Noggin secretion by PC-3/F*luc* cells and by the different clones was determined in concentrated CM, normalized by the total cell number collected from each sample. Proteins were separated on 12% sodium dodecyl sulfate-polyacrylamide gels (Mini Protean Gel BioRad) and transferred on Hybond-P membranes (GE Healthcare, Glattburg, Switzerland). Membranes were incubated overnight with 40 ng/ml of a rat monoclonal antibody against the human native noggin protein [20] (RP57-16; kindly provided by Dr. A.N. Economides, Regeneron Pharmaceuticals, Inc.) and detected with a horseradish peroxidase-labeled anti-rat secondary antibody (1∶1000; GE Healthcare). Immunoreactivity was visualized with the ECL Advanced chemiluminescence substrate (GE Healthcare) using the VersaDoc imaging system (Bio-Rad Laboratories, Reinach, Switzerland) and signal intensity was quantified with QuantityOne imaging software (Bio-Rad Laboratories). Equivalent protein loading was verified by staining with Coomassie blue the total protein content in the CM.

### Intra-osseous inoculation of PC-3/F*luc* cells, mock and Nog-KD clones

The cells were inoculated into the marrow cavity of the left tibia at a concentration of 5×10^5^/10 µl of phosphate-buffered saline (PBS), as previously described [18]. Groups of seven animals each were inoculated with either PC-3/F*lu*c cells, mock or Nog-KD clones. Another group of 7 animals, inoculated with phosphate-buffered saline alone served as control ( =  sham). In a first experiment tumor growth was allowed to progress until day 25, at which time animals from all groups were sacrificed because of the severe osteolysis and imminent risk of fracture in the PC-3/F*lu*c and mock groups. In a further experiment the animals xenografted with the PC-3/F*lu*c clone were sacrificed at day 23 for the same reason above, while the groups xenografted with Nog-KD 14 and 17 clones were kept for a period of 30 and 33 days, respectively. The xenografted and sham-operated tibiae were dissected and fixed in 4% paraformaldehyde (PFA).

### Intra-cardiac injection of PC-3/F*luc* cells, mock and Nog-KD clones

The cells were injected into the left cardiac ventricle at the concentration of 10^5^/100 µl in PBS, as previously described [18]. Groups of ten animals each were injected with either PC-3/F*lu*c or mock 4 or Nog-KD 17 clones. Animals were sacrificed 28 days after injection.

### Radiography

Radiographs of the mice were taken weekly using a Faxitron radiography system (MX-20, Faxitron X-Ray Corporation, Edimex, Le Plessis, France) to monitor changes in bone structure and radiodensity.

### *In vivo* bioluminescent imaging (BLI)

Tumor growth was monitored non-invasively in the living animals by bioluminescent imaging (BLI) at weekly intervals as previously described [18] using an ultrasensitive charge-coupled device (CCD) camera (NightOWL LB, Berthold Technologies, Bad Wilbad, Germany). *In vivo* photon counts were normalized for the *in vitro* luciferase activity by PC-3/F*lu*c clone, mock and Nog-KD clones.

### Three-dimensional micro-computer tomography (µ-CT) of bone lesions

To obtain qualitative and quantitative assessment of the tumor-induced modifications of the bone structure, sham and tumor-bearing tibiae were scanned *ex vivo*, at the end of the experimental period, with a µ-CT40 scanner (SCANCO Medical AG, Brüttisellen, Switzerland) at a resolution of 6 µm. The region of interest was selected from the scout view and 1600 micro-tomographic slices were acquired, covering three quarters of the length of the tibia. Tridimensional images were obtained through reconstruction of cross sectional images. Quantitative analysis of primary parameters such as total volume (TV) and bone volume (BV) was performed on a volume of interest equivalent to 166 slides (1 mm in length) at approximately 2.5 mm distal from the cleft of the knee joint as reference point.

### Peripheral quantitative computed tomography (pQCT)

Bone mineral content in the xenografted tibiae was determined *ex vivo* with a small animal pQCT scanner (XCT Research SA; Norland Stratec, Pforzheim, Germany) at the end of the experimental period. Measurements were performed at 2 and 3 mm distal from the reference point (cleft of the knee joint).

### Bone histomorphometry

Fixed tibiae were decalcified in a solution of PFA 0.5%/EDTA 15% for 20 days and processed for paraffin embedding. Four-µm sections were stained for tartrate-resistant acid phosphatase (TRAP) as described previously [21], counterstained with hematoxylin and examined by light microscopy. Osteoclasts were counted as multinucleated TRAP-positive cells on the endosteal surface of cortical and trabecular bone in sham-operated tibiae, and on the residual bone adjacent to tumor cells in cancer cell-xenografted tibiae. The number of osteoclasts (N.Oc/BS; /mm) and of active osteoblasts (N.Ob/BS; /mm), the bone surface covered by osteoclasts (Oc.S/BS; %) and osteoblasts (Ob.S/BS; %) were determined using the OsteoMeasure histomorphometry software (OsteoMetrics Inc., Decatur, GA, USA). Osteoclasts and osteoblasts were counted in randomly selected fields of an area >1 mm^2^, approximately at 1.5 mm from the growth plate. Bone parameters were expressed as recommended in the report of the ASBMR Histomorphometry nomenclature committee [22].

### Statistical analysis

Graph Pad Prism version 4.00 for Windows (GraphPad Software, San Diego, CA, USA) was used for all statistical analyses. The parametric one-way ANOVA test with a Bonferroni post-test was used to analyze the RNA expression data, the bone architecture parameters obtained by µ-CT and pQCT, and the histomorphometric data. To compare the cell proliferation rate *in vitro* and *in vivo* the parametric two-way ANOVA test was employed. *P* values smaller than or equal to 0.05 were considered as significant.

## Supporting Information

Figure S1 **Luciferase expression does not modify the osteolytic potential of PC-3 cells and moderately affects their gene expression** ***in vitro*** **.** **A**. Representative images of radiography of tibiae xenografted with PC-3 or PC-3/F*luc* cells at day 28 after intra-osseous inoculation. **B**. Expression of noggin, Dkk-1, PTHrP, RANKL, CSF-1 and IL-8 mRNA. mRNA expression levels (+/− SD) are quantified by real-time RT-PCR and normalized to β-actin as endogenous control. mRNA expression level in PC-3 cells is set as 100%; the mean of 2 to 3 independent experiments is shown. ***P<0.001, PC-3/F*luc versus* PC-3 cells.
(TIF)

Figure S2 **Noggin silencing promotes increase in radiodensity in advanced osteolytic lesions.** Radiographic aspect of sham-operated and cancer cell-xenografted tibiae at day 21 after intra-osseous inoculation.
(TIF)

Figure S3 **Noggin silencing promotes partial bone repair in advanced osteolytic lesions and limits late tumor growth.** **A**. Radiographic aspect and **B**. 3-D reconstruction (µ-CT) of tibiae xenografted with PC-3/F*luc* and Nog-KD clones, at day 23 (PC-3/F*luc*), day 30 (Nog-KD 17) and day 33 (Nog-KD 14) after inoculation. **C**. Growth *in vivo* of PC-3/F*luc* and Nog-KD clones. Bioluminescent signal (photon counts +/− SD) emitted from the cancer cell-xenografted tibiae was quantified at day 7, 14, 21, 23, 28, 30 and 33 after intra-osseous implantation of tumor cells; *n*  =  6–7 animals for each experimental group. ***P<0.001, Nog-KD clones *versus* PC-3/F*luc* at day 21.
(TIF)

Table S1 The sequence of the different shRNA obtained from Superarray is shown.
(DOC)

Table S2 Assay ID of the real-time primers and probes obtained from Applied Biosystems are listed.
(DOCX)
