# Supplementary figures and images for: The Role of the BMP Signaling Antagonist Noggin in the Development of Prostate Cancer Osteolytic Bone Metastasis
Source: PLoS One. 2011 Jan 13;6(1):e16078. doi: 10.1371/journal.pone.0016078 (PMC3020964; doi:10.1371/journal.pone.0016078)

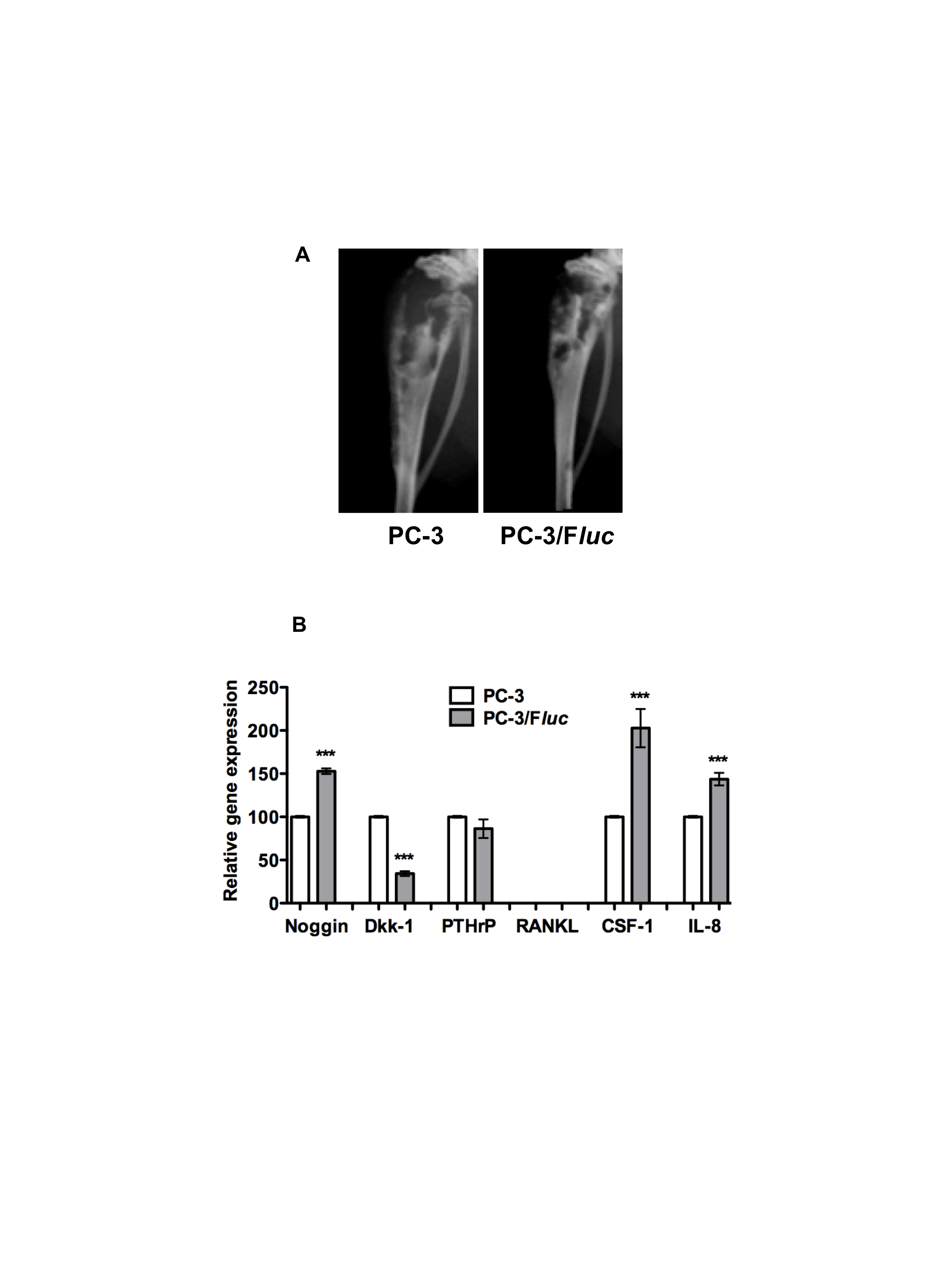

Supplement: Figure S1 — Luciferase expression does not modify the osteolytic potential of PC-3 cells and moderately affects their gene expression in vitro . A. Representative images of radiography of tibiae xenografted with PC-3 or PC-3/Fluc cells at day 28 after intra-osseous inoculation. B. Expression of noggin, Dkk-1, PTHrP, RANKL, CSF-1 and IL-8 mRNA. mRNA expression levels (+/− SD) are quantified by real-time RT-PCR and normalized to β-actin as endogenous control. mRNA expression level in PC-3 cells is set as 100%; the mean of 2 to 3 independent experiments is shown. ***P<0.001, PC-3/Fluc versus PC-3 cells. (TIF) [file pone.0016078.s001.tif]

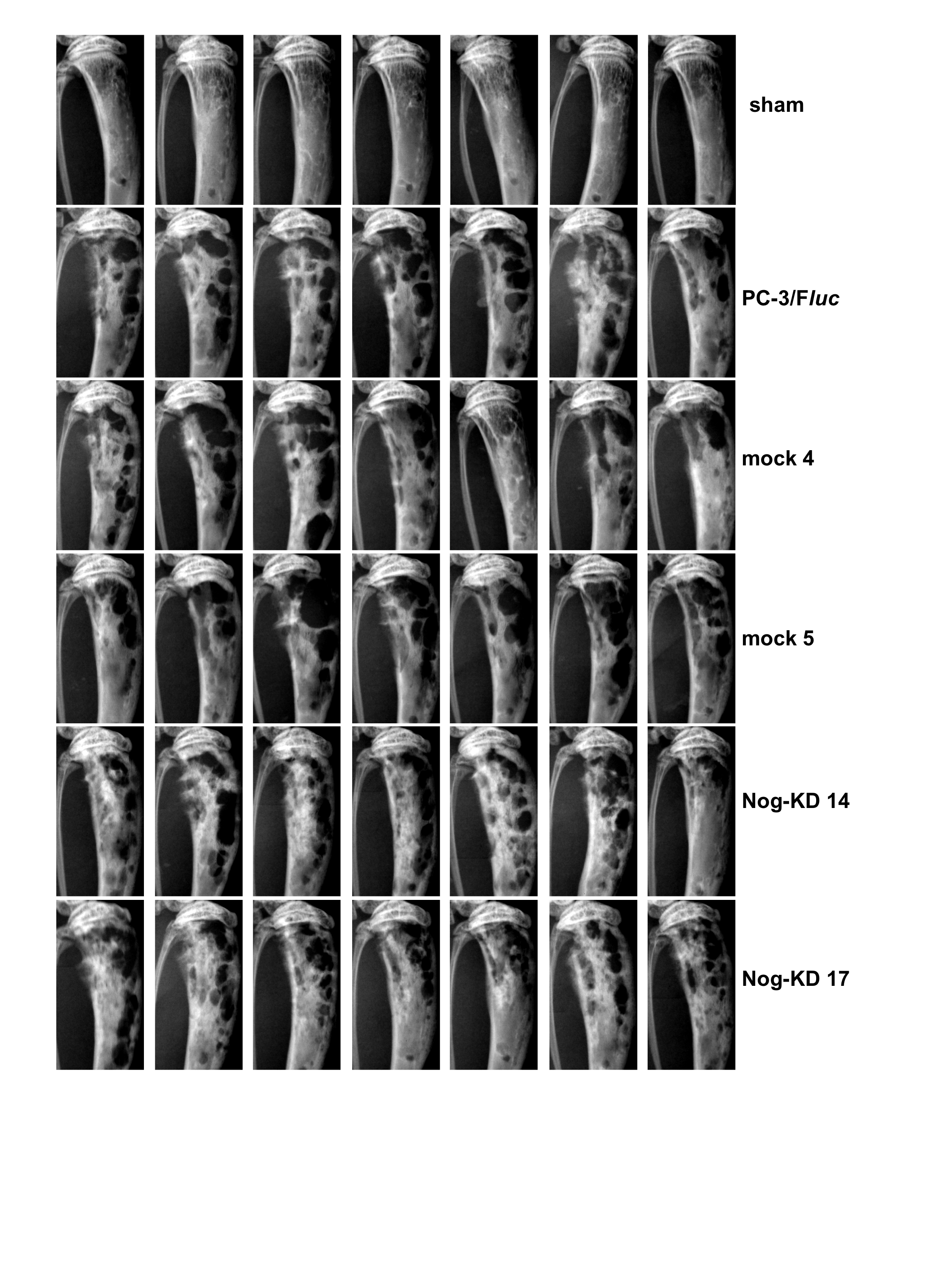

Supplement: Figure S2 — Noggin silencing promotes increase in radiodensity in advanced osteolytic lesions. Radiographic aspect of sham-operated and cancer cell-xenografted tibiae at day 21 after intra-osseous inoculation. (TIF) [file pone.0016078.s002.tif]

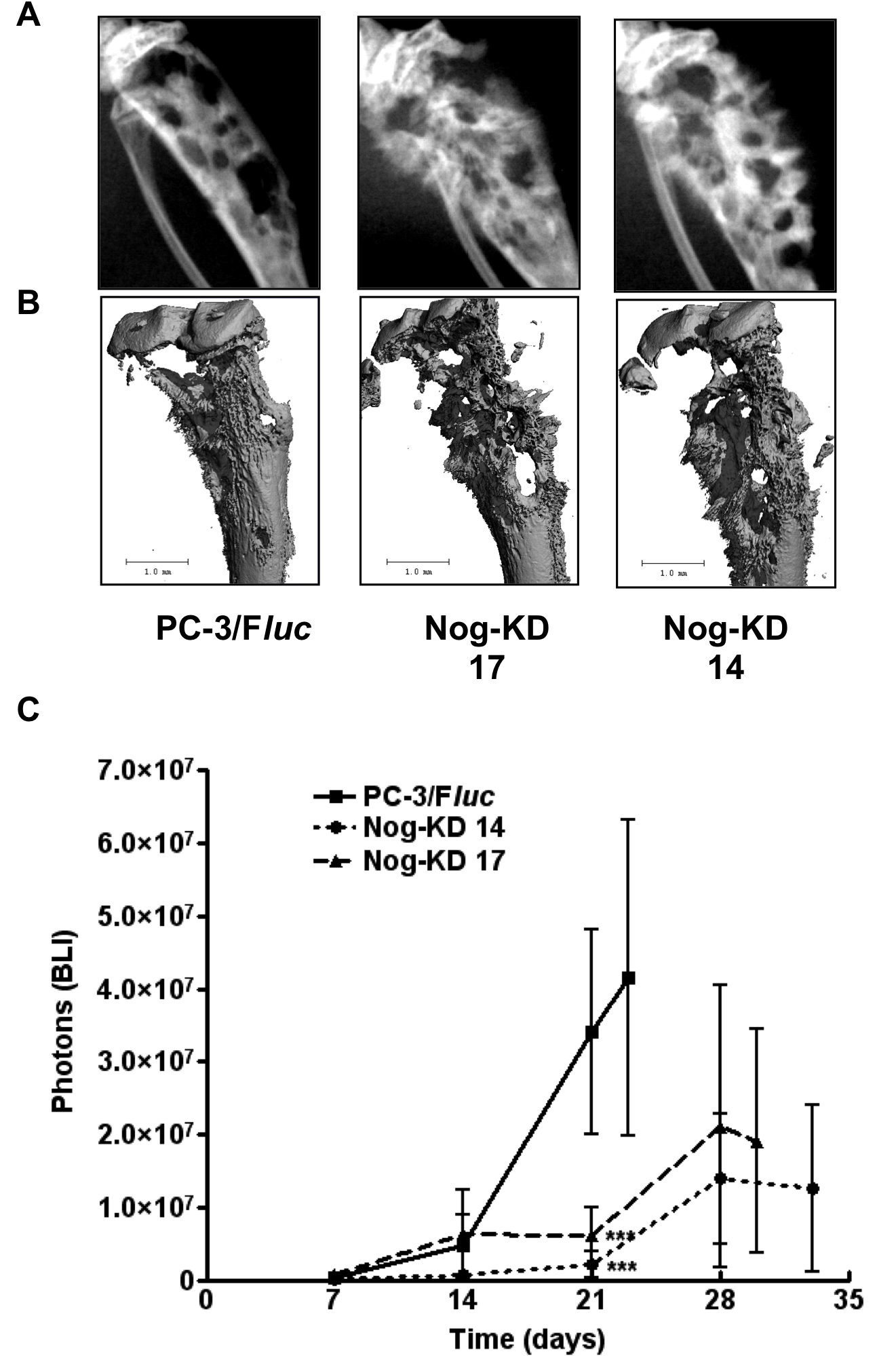

Supplement: Figure S3 — Noggin silencing promotes partial bone repair in advanced osteolytic lesions and limits late tumor growth. A. Radiographic aspect and B. 3-D reconstruction (µ-CT) of tibiae xenografted with PC-3/Fluc and Nog-KD clones, at day 23 (PC-3/Fluc), day 30 (Nog-KD 17) and day 33 (Nog-KD 14) after inoculation. C. Growth in vivo of PC-3/Fluc and Nog-KD clones. Bioluminescent signal (photon counts +/− SD) emitted from the cancer cell-xenografted tibiae was quantified at day 7, 14, 21, 23, 28, 30 and 33 after intra-osseous implantation of tumor cells; n = 6–7 animals for each experimental group. ***P<0.001, Nog-KD clones versus PC-3/Fluc at day 21. (TIF) [file pone.0016078.s003.tif]
