# Supplementary material for: The Role of the BMP Signaling Antagonist Noggin in the Development of Prostate Cancer Osteolytic Bone Metastasis
Source: PLoS One. 2011 Jan 13;6(1):e16078. doi: 10.1371/journal.pone.0016078 (PMC3020964; doi:10.1371/journal.pone.0016078)
Supplement: Table S1 — The sequence of the different shRNA obtained from Superarray is shown. (DOC) [file pone.0016078.s004.doc]

**Supportive Table 1.** **Sequences of the different shRNA used in this study.**

| **shRNA construct*** | **Insert sequence** |
| --- | --- |
| Noggin sh-RNA 1 | GCTACAGAGACCTAGCTTTCT |
| Noggin sh-RNA 2 | GTGTATATACGGTCCCAGTTT |
| Noggin sh-RNA 3 | TGTGTATATACGGTCCCAGTT |
| Non targeting-shRNA | GGAATCTCATTCGATGCATAC |

* shRNA constructs were purchased from Superarray Biosciences
