# Supplementary material for: The Role of the BMP Signaling Antagonist Noggin in the Development of Prostate Cancer Osteolytic Bone Metastasis
Source: PLoS One. 2011 Jan 13;6(1):e16078. doi: 10.1371/journal.pone.0016078 (PMC3020964; doi:10.1371/journal.pone.0016078)
Supplement: Table S2 — Assay ID of the real-time primers and probes obtained from Applied Biosystems are listed. (DOCX) [file pone.0016078.s005.docx]

**Supportive Table 2.** **Real-time primers and probes used in this study.**

| **Gene** | **Primer/probe*** |
| --- | --- |
| ß-actin | Hs_99999903_m1 |
| Noggin | Hs_00271352_s1 |
| Dkk-1 | Hs_00183740_m1 |
| PTHrP | Hs_00174969_m1 |
| CSF-1 | Hs_00174164_m1 |
| RANKL | Hs_00243522_m1 |
| IL-8 | Hs_00184979_m1 |
| BMP-2 | Hs_00154192_m1 |
| BMP-3 | Hs_00609638_m1 |
| BMP-4 | Hs_00181626_m1 |
| BMP-6 | Hs_00233470_m1 |
| BMP-7 | Hs_00233477_m1 |

* TaqMan assays-on-demand were from Applied Biosystems
